# Palmitic Acid, but Not Lauric Acid, Induces Metabolic Inflammation, Mitochondrial Fragmentation, and a Drop in Mitochondrial Membrane Potential in Human Primary Myotubes

**DOI:** 10.3389/fnut.2021.663838

**Published:** 2021-05-31

**Authors:** Domenico Sergi, Natalie Luscombe-Marsh, Nenad Naumovski, Mahinda Abeywardena, Nathan O'Callaghan

**Affiliations:** ^1^Nutrition and Health Program, Health and Biosecurity, Commonwealth Scientific and Industrial Research Organisation, Adelaide, SA, Australia; ^2^Adelaide Medical School, The University of Adelaide, Adelaide, SA, Australia; ^3^Diabetes SA, Adelaide, SA, Australia; ^4^Faculty of Health, University of Canberra, Canberra, ACT, Australia; ^5^Functional Foods and Nutrition Research Laboratory, University of Canberra, Bruce, ACT, Australia

**Keywords:** fatty acids, mitochondria, metabolic inflammation, skeletal muscle, lauric acid, palmitic acid

## Abstract

The chain length of saturated fatty acids may dictate their impact on inflammation and mitochondrial dysfunction, two pivotal players in the pathogenesis of insulin resistance. However, these paradigms have only been investigated in animal models and cell lines so far. Thus, the aim of this study was to compare the effect of palmitic (PA) (16:0) and lauric (LA) (12:0) acid on human primary myotubes mitochondrial health and metabolic inflammation. Human primary myotubes were challenged with either PA or LA (500 μM). After 24 h, the expression of *interleukin 6 (IL-6)* was assessed by quantitative polymerase chain reaction (PCR), whereas Western blot was used to quantify the abundance of the inhibitor of nuclear factor κB (IκBα), electron transport chain complex proteins and mitofusin-2 (MFN-2). Mitochondrial membrane potential and dynamics were evaluated using tetraethylbenzimidazolylcarbocyanine iodide (JC-1) and immunocytochemistry, respectively. PA, contrarily to LA, triggered an inflammatory response marked by the upregulation of *IL-6* mRNA (11-fold; *P* < 0.01) and a decrease in IκBα (32%; *P* < 0.05). Furthermore, whereas PA and LA did not differently modulate the levels of mitochondrial electron transport chain complex proteins, PA induced mitochondrial fragmentation (37%; *P* < 0.001), decreased MFN-2 (38%; *P* < 0.05), and caused a drop in mitochondrial membrane potential (11%; *P* < 0.01) compared to control, with this effect being absent in LA-treated cells. Thus, LA, as opposed to PA, did not trigger pathogenetic mechanisms proposed to be linked with insulin resistance and therefore represents a healthier saturated fatty acid choice to potentially preserve skeletal muscle metabolic health.

## Introduction

There is an exponential increase in the incidence of obesity and type 2 diabetes mellitus (T2DM) in the developed and developing countries, (1). Individuals affected by T2DM are at higher risk of developing severe health consequences including cardiovascular disease, nephropathy, and neuropathy (2). Furthermore, this metabolic disorder is also emerging as a risk factor for the development of neurodegenerative diseases including Parkinson and Alzheimer disease (3, 4). T2DM is characterised by chronic hyperglycaemia, which is a direct consequence of insulin resistance and pancreatic β-cell dysfunction. Particularly, insulin resistance is the hallmark of T2DM and develops in the skeletal muscle decades before pancreatic β-cells become dysfunctional and overt hyperglycaemia develops (5). Therefore, skeletal muscle insulin resistance represents a primary defect in the pathogenesis of T2DM. In terms of the mechanisms underpinning the onset of skeletal muscle insulin resistance, mitochondrial dysfunction and metabolic inflammation have been reported to play a pivotal role (6–8). Mitochondrial dysfunction in the face of increased fatty acid availability promotes the buildup of lipotoxic lipid species, such as ceramides, which disrupt insulin signalling pathway by activating PKC isoform protein kinase Cζ and protein phosphatase 2A, which in turn directly hamper AKT (protein kinase B) activation (9). With regard to metabolic inflammation, the activation of proinflammatory nuclear factor κ light-chain enhancer of activated B cells (NFκB) signalling and c-Jun N-terminal kinases (JNKs) have been reported to impede insulin signal transduction pathway, underpinned by the serine phosphorylation of insulin receptor substrate (10, 11).

Mitochondria bioenergetics and inflammation, apart from being involved in skeletal muscle insulin resistance, are also interrelated processes, with metabolic inflammation being able to impair mitochondrial function and mitochondria themselves, contributing to the activation of intracellular proinflammatory responses (12, 13). Particularly, the activation of the proinflammatory NFκB signalling in response to nutrient overload has been reported to promote mitochondrial dysfunction with a shift in mitochondrial dynamics towards fission (12). Nonetheless, mitochondrial dysfunction may also trigger the activation of proinflammatory pathways by promoting the accumulation of lipotoxic lipid intermediates (14, 15) or *via* the release of damage-associated molecular patterns such as mitochondrial DNA or increase production of reactive oxygen species (13).

Circulating nutrient excess and particularly the overconsumption of long-chain saturated fatty acids have been reported to trigger metabolic inflammation in a variety of tissues (15–19), as well as mitochondrial dysfunction marked by mitochondrial fission, decreased oxidative capacity, and increased production of reactive species (8, 20–22). These effects have been well-described for palmitic acid (PA), with this long-chain saturated fatty acid being reported to induce mitochondrial dysfunction and metabolic inflammation in a variety of tissues both in the central nervous system and the periphery (12, 23–27). The induction of these pathogenetic mechanisms culminates with the onset of insulin resistance and impaired metabolic health (24, 28). However, not all the saturated fatty acids have been reported to be metabolically detrimental. Indeed, medium-chain saturated fatty acids, such as lauric acid (LA), have been shown not to promote insulin resistance, to be less obesogenic, and to decrease the ratio of total cholesterol to high-density lipoprotein cholesterol compared to longer chain saturated fatty acids (29–32). LA has been reported to be less metabolically detrimental and proinflammatory compared to PA (33). Additionally, LA, as opposed to PA, is β-oxidised more effectively, which, in turn, may prevent the deleterious effects linked with intramyocellular lipid accumulation and lipotoxicity (34). Despite accumulating evidence on the metabolic effect of medium-chain saturated fatty acids on metabolic health, their effect on human skeletal muscle and particularly their ability to modulate inflammatory responses and mitochondrial health remain to be elucidated. Indeed, the studies conducted to date, to our knowledge, reported on the effect of medium-chain fatty acids on mitochondrial function in rodents and cell lines (35, 36); there is therefore a lack of data on human tissues. Furthermore, the activation of proinflammatory NFκB signalling has been shown to trigger mitochondrial fragmentation (12); therefore, fatty acids differing for their ability to trigger the activation of this signalling pathway should also differently affect mitochondrial health.

Thus, in consideration of the lack of evidence in experimental models closely mimicking human physiology and considering the putatively less harmful metabolic effect of LA compared to PA, the aim of the present study was to compare the effect of PA and LA on key mechanisms governing in metabolic health. Particularly, we aimed at investigating the effects of these long- and medium-chain saturated fatty acids on mitochondrial dynamics, mitochondrial electron transport chain complex proteins, mitochondrial membrane potential, and metabolic inflammation in human primary myotubes.

## Materials and Methods

### Human Primary Myoblasts and Reagents

Human primary myoblasts from four different donors were obtained from Cook Myosite (USA). Dulbecco modified eagle low glucose medium (DMEM) containing 5.5 mM glucose, horse serum (HS), heat-inactivated foetal bovine serum (FBS), penicillin–streptomycin solution (10,000 U/ml), Pierce™ BCA protein assay kit, TaqMan assays, TaqMan fast advanced master mix, and rabbit anti–mouse immunoglobulin G (IgG) secondary antibody (Alexa Fluor 488) were from Life Technologies Australia Pty. Ltd. (Mulgrave, Victoria, Australia). The primary antibodies, anti-inhibitor of nuclear factor κB (IκBα), anti–MFN-2, anti–β-actin, and the secondary antibodies, anti–rabbit IgG horseradish peroxidase (HRP)–linked antibody and anti–mouse IgG HRP-linked antibody, were obtained from Cell Signalling Technology. JC-1 mitochondrial membrane potential assay kit, total OXPHOS rodent antibody cocktail, and anti-TOMM20 were from Abcam Australia Pty. Ltd. (Melbourne, Victoria, Australia). PA and LA, as well as low-endotoxin fatty acid–free bovine serum albumin (BSA), were purchased from Sigma–Aldrich (Castle Hill, New South Wales, Australia). Finally, Trans-Blot Turbo Midi 0.2 μm polyvinylidene fluoride (PVDF) Transfer Packs and 4 to 15% Criterion TGX Precast Midi Protein Gel were obtained from Bio-Rad (Gladesville, New South Wales, Australia).

### Cell Cultures and Fatty Acid Treatments

Human primary myoblasts derived from the abdominal rectus muscles of four non-type 2 diabetic, non-obese male individuals 31.00 ± 5.67 years of age, body mass index 24.75 ± 1.31 kg/m^2^ (Human skMDC; Cook Myosite) were maintained in DMEM (low glucose) containing 20% FBS and 100 U/ml penicillin–streptomycin at 37°C under a 5% CO_2_ atmosphere as previously reported (37). Cells were grown in 25-cm^2^ flasks, 6-, 24-, or 96-well plates for the assessment gene expression, protein expression by Western blot, immunocytochemistry, or assessment of mitochondrial membrane potential, respectively. Myoblast differentiation was initiated when cells reached 75 to 80% confluence by incubating the cells for 7 days in DMEM containing 2% HS and 100 U/ml penicillin–streptomycin. After 7 days, cells were treated with either low-endotoxin fatty acid–free BSA, 500 μM PA, or 500 μm LA for 24 h in differentiation media, and then samples collected for gene or protein expression or directly assayed for changes in mitochondrial membrane potential and morphology. The concentration of PA used in the present study falls within the circulating physiological range, as previously reported (38). Hence, to compare the effect of PA and LA, these fatty acids were used at the same concentrations to allow for an equimolar comparison.

### Fatty Acid Conjugation to BSA

Fatty acids are fat-soluble molecules and therefore insoluble in the cell culture media in their native form; furthermore, free fatty acids travel in the bloodstream bound to albumin. Thus, to allow for fatty acid solubilisation in the cell culture media and mimic fatty acid physiological circulating conditions, both PA and LA were conjugated to BSA as described previously (15). Low-endotoxin BSA was used to prevent potential lipopolysaccharide contamination as reported previously (39). Fatty acids were dissolved in 0.1 M NaOH in a water bath at 70°C to yield a final concentration of 20 mM. BSA was solubilised in serum and penicillin-streptomycin–free DMEM at 55°C and fatty acid mixed with the fatty acid solutions to obtain a 1:4 molar ratio (fatty acids 2 mM: BSA 0.5 mM). The fatty acid–BSA mix was vortexed and then incubated for 10 min at 55°C and cooled to room temperature before being filter sterilised. Conjugated fatty acids were stored at −20°C before use.

### RNA Extraction, cDNA Synthesis, and Assessment of Gene Expression by Real-Time PCR–PCR

After the fatty acid treatments, cells were directly lysed in the 25-cm^2^ flasks using RLT lysis buffer (Qiagen, Chadstone, Victoria, Australia), cell lysates were collected, and total RNA extracted using RNeasy Mini Kit (Qiagen) according to the manufacturer instructions. cDNA was synthesised starting from 0.5 μg of total RNA as a template and using SuperScript II (Life Technologies Australia Pty. Ltd.) as described previously (40). Real-time PCR to assess the expression of *IL-6* and *ribosomal protein S18* was carried out using CFX Connect 96 real-time PCR detection system (Bio-Rad) employing a two-step cycling program of 95°C for 20 s, and then 40 cycles at 95°C for 3 s and 60°C for 30 s. Each reaction contained 1 μl of cDNA template, 5 μl of TaqMan fast advanced master mix, 3.5 μl of diethylpyrocarbonate-treated water, and 0.5 μl of either of the following Taqman assays: *ribosomal protein S18* Hs01375212_g1 and *IL-6*: Hs00174131_m1. Ct values were normalised to the reference gene *ribosomal protein S18* and data analysed using the comparative ΔCt method (41).

### Western Blot

Following 24-h incubation with BSA or fatty acids, cells were washed with phosphate-buffered saline (PBS) and directly lysed with RIPA buffer (Sigma–Aldrich) containing Halt Protease and Phosphatase Inhibitor Cocktail (Life Technologies Australia Pty. Ltd.). Cell lysate proteins were quantified using Pierce™ BCA protein assay kit and 6 μg of protein loaded and resolved on a 4 to 15% precast polyacrylamide gel. Proteins were then transferred onto PVDF membranes using a Trans-Blot Turbo Transfer System (Bio-Rad). Non-specific binding sites were blocked by incubating the membranes for 1 h at room temperature with a 5% BSA solution made up in PBS. Membranes were then probed overnight with total OXPHOS rodent antibody cocktail, anti-IκBα, anti–MFN-2, and anti–β-actin followed by three 5-min washes with PBS containing 0.1% Tween-20 (PBST). After the washes, membranes were incubated with HRP-linked secondary antibodies as appropriate. Membranes were finally washed three times for 5 min in PBST before being revealed using Western lightning ECL Pro (PerkinElmer). Western blot bands intensities were quantified by densitometric analysis using NIH ImageJ software (NIH, USA).

### Immunocytochemistry and Assessment of Mitochondrial Morphology

Cells were grown and differentiated onto sterile glass coverslips placed in 24-well plates. After differentiation, myotubes were challenged with either BSA, PA, or LA followed by fixation with 4% formaldehyde for 20 min at room temperature. Cells were then washed with PBS and permeabilised using a 0.5% triton X PBS solution for 15 min followed by incubation with 2% BSA dissolved in PBS containing 0.25% Triton X (blocking solution) in order to block non-specific bindings. After permeabilisation and blocking, cells were incubated for 2 h at room temperature with anti-TOMM20 diluted 1:1,000 in permeabilisation solution and then washed with PBS before being incubated with rabbit anti–mouse IgG secondary antibody (Alexa Fluor 488) for 1 h at room temperature in the dark. Incubation with secondary antibody was followed by three washes with PBS. Finally, coverslips were mounted on slides using fluoroshield with DAPI to counterstain the nuclei. Cells were imaged using the EVOS imaging system (Thermo Fisher Scientific, Bothell, WA, USA). Mitochondria morphology was evaluated using the ImageJ macro developed by Dagda et al. (42), as described previously (43). The circularity score was used to define mitochondria morphology, with an increase in the score indicating a higher degree of fragmentation.

### Measurement of Mitochondrial Membrane Potential

JC-1 mitochondrial membrane potential assay kit (Abcam Australia Pty. Ltd.) was used to measure mitochondrial membrane potential. JC-1 is a cationic fluorescent dye that accumulates within the mitochondria in a membrane potential-dependent manner. After 24-h exposure to either BSA, PA, or LA, JC-1 was directly administered directly to cell culture media at a final concentration of 10 μM and cells incubated for 10 min at 37°C in the dark. Cells were then washed three times with PBS and JC-1 red fluorescence recorded at 550/615 (excitation/emission) and green fluorescence at 489/535 (excitation/emission) using Victor3 V 1420 Multilabel Counter (PerkinElmer). Finally, the ratio of red to green fluorescence was calculated to quantify mitochondrial membrane potential. The green fluorescence quantified at 489/535 (excitation/emission) is directly proportional to mitochondrial membrane potential, whereas red fluorescence, detected at 550/615 (excitation/emission), represents JC-1 aggregates that accumulate within the mitochondria when membrane potential exceeds −240 mV (44). In light of this, calculating the ratio between red vs. green fluorescence allows the quantification of mitochondrial membrane potential independently of the number of mitochondria assessed. After fluorescence quantification, cells were imaged using the EVOS imaging system to obtain representative images (Thermo Fisher Scientific).

### Statistical Analysis

Data are expressed as mean ± SEM and represent the experiments conducted on cells derived from four independent individuals (*n* = 4) and analysed at least in duplicate as reported in figure legends. Difference between treatments was assessed using repeated-measures one-way analysis of variance followed by Tukey *post hoc* test using GraphPad Prism 8 for Windows. *p* < 0.05 was considered statistically significant.

## Results

### PA, but Not LA, Triggers an Inflammatory Response in Human Primary Myotubes

**Figure 1:**
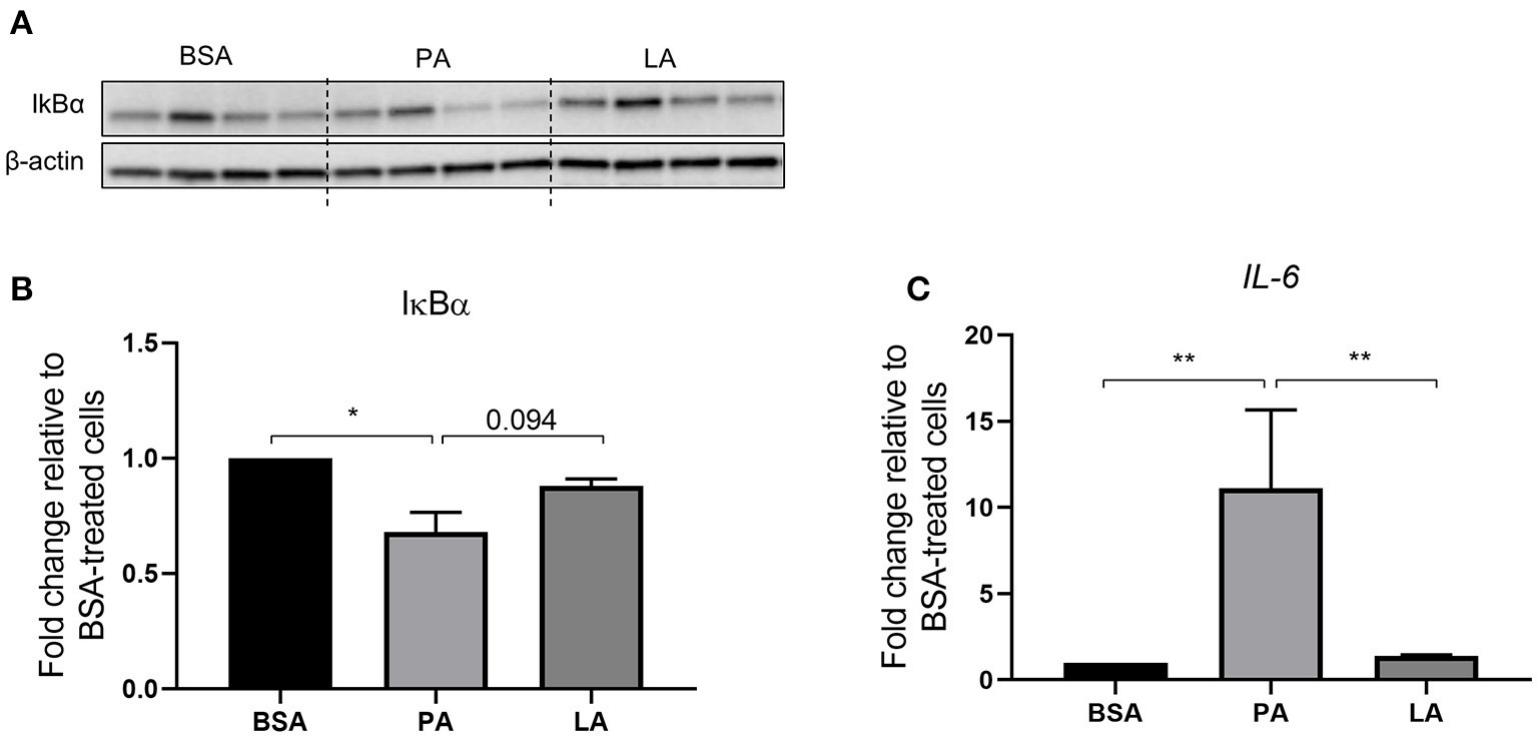
Activation of the IKKβ-NFκB signalling pathway in human primary myotubes in response to saturated fatty acids. **(A)** Representative Western blot of cellular IκBα abundance, **(B)** densitometric analysis of IκBα normalised to β-actin, and **(C)** gene expression analysis of *IL-6* by quantitative PCR. Data are reported as means ± SEM, *n* = 4 cultures from independent donors (means of two replicates). ^*^P < 0.05, ^**^P < 0.01.

Metabolic inflammation and particularly the activation of the NFκB signalling pathway was investigated in order to assess the difference between PA and LA in their ability to trigger an inflammatory response. PA decreased the abundance of IκBα (*p* < 0.05) (Figures 1A,B), a protein that forms a stable complex with NFκB and inhibits its transcriptional activity. However, upon activation of the proinflammatory NFκB signalling, IκBα is phosphorylated by IκB kinase (IKK) and channelled towards proteasomal degradation, thus allowing NFκB to migrate to the nucleus and induce the expression of its gene targets (45). In light of this, PA-induced decrease in IκBα marks the activation of the NFκB signalling pathway, which was further confirmed by the upregulation of *IL-6*, a NFκB target gene (46), compared to BSA and LA-treated cells (*p* < 0.01) (Figure 1C). However, despite LA also being a saturated fatty acid, it did not decrease IκBα relative to BSA (*p* > 0.05) while tended to induce its abundance compared to PA (*p* = 0.094). Furthermore, LA did not increase *IL-6* expression compared to BSA-treated cells (Figure 1C).

### Both PA and LA Modulate the Abundance of Mitochondrial Electron Transport Chain Proteins

**Figure 2:**
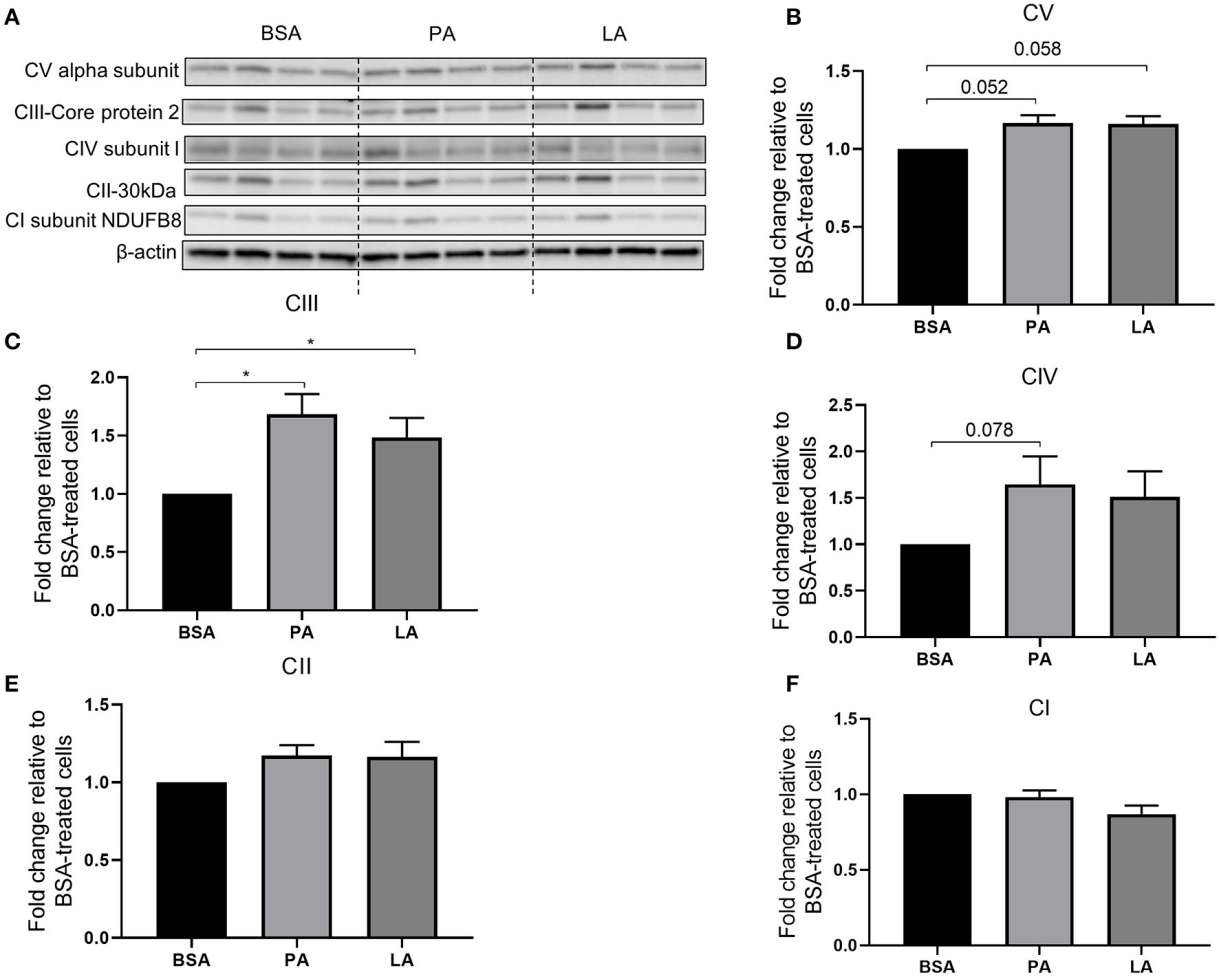
Relative abundance of electron transport chain complex proteins in response to palmitic or lauric acid. **(A)** Representative Western blot of CV alpha subunit (complex V), CIII-Core protein 2 (complex III), CIV subunit I (complex IV), CII-30kDa (complex II), CI subunit NDUFB8 (complex I). Densitometric analysis normalised to β-actin of CV alpha subunit (complex V) **(B)**; CIII-Core protein 2 (complex III) **(C)**; CIV subunit I (complex IV) **(D)**; CII-30kDa (complex II) **(E)**; CI subunit NDUFB8 (complex I) **(F)**. Data are reported as means ± SEM, *n* = 4 cultures from independent donors (means of two replicates). ^*^P < 0.05.

Mitochondria are key organelles for the catabolism of fatty acids; therefore, the relative abundance of mitochondrial electron transport chain complex proteins was assessed to evaluate whether PA and LA differently modulate their expression. Both PA and LA tended to induce CV alpha subunit (complex V) (*p* = 0.052 and *p* = 0.058, respectively) (Figures 2A,B) and upregulated CIII-Core protein 2 (complex III) (*p* < 0.05) (Figures 2A,C). Furthermore, while PA tended to upregulate CIV subunit I (complex IV) (*p* = 0.078) compared to BSA (Figures 2A,D), LA did not affect the abundance of this protein (Figures 2A,D). Finally, neither PA nor LA induced the abundance of CII-30 kDa (complex 2) (Figures 2A,E), nor CI subunit NDUFB8 (complex I) (Figures 2A,F).

### PA Promotes Mitochondrial Fragmentation

**Figure 3:**
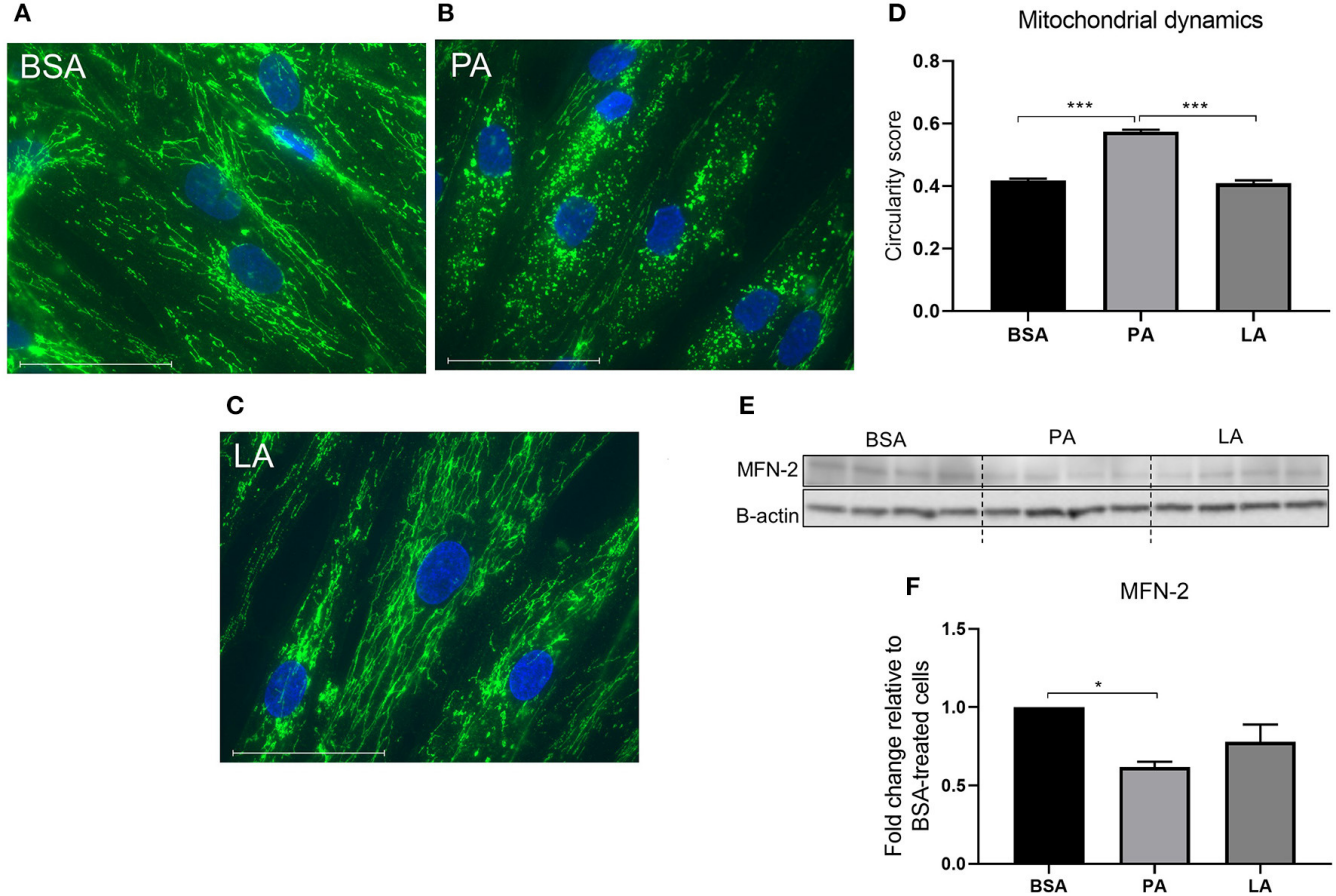
The effect of palmitic and lauric acid on mitochondrial dynamics. Myotubes were immunostained with anti-TOMM20 and fluorescence detected using the EVOS imaging system following 24 incubation with BSA **(A)**, PA **(B)**, and LA **(C)**. Nuclei were counterstained with DAPI (blue). Scale bar = 10 μm. **(B)** PA induced mitochondrial fragmentation. **(D)** Image analysis of fluorescence-labelled mitochondria, values represent mitochondrial circularity score, with a higher score indicating increased mitochondrial circularity and fragmentation. **(E)** Representative Western blot reflecting the abundance of MFN-2. **(F)** Densitometric analysis of MFN-2 normalised to β-actin. Data are reported as means ± SEM, *n* = 4 cultures from independent donors. Image analysis was performed on 10 different fields per treatment on each independent cell line. Western blot data are the means of three replicates. *P < 0.05, ***P < 0.001.

Mitochondria go through cycles of fusion and fission, termed *mitochondrial dynamics*. Mitochondrial dynamics represents a key modulator of mitochondrial oxidative capacity, allowing the cells to adapt to short-term changes in fuel availability (47). PA induced an increase in mitochondrial fragmentation as indicated by a drop in the circularity score compared to BSA-treated cells (*p* < 0.001) (Figures 3A,B,D). However, LA, despite being a saturated fatty acid, did not induce mitochondria fragmentation (Figures 3C,D), with LA-treated myotubes maintaining a tubular mitochondrial network comparable to cells challenged with PA (Figure 3C). MFN-2 is a key protein responsible for promoting mitochondrial fusion (48) with its overexpression ameliorating PA-induced insulin resistance (49). Therefore, to further confirm the effect of PA on mitochondria dynamics, its expression was assessed in response to fatty acid challenges. While PA downregulated MFN-2 compared to myotubes treated with BSA (*p* < 0.05) (Figures 3E,F), the same effect was not elicited by LA (Figures 3E,F), confirming that PA and LA differently regulate mitochondrial dynamics, with PA promoting a shift towards fission.

### PA and LA Differently Regulate Mitochondrial Membrane Potential

**Figure 4:**
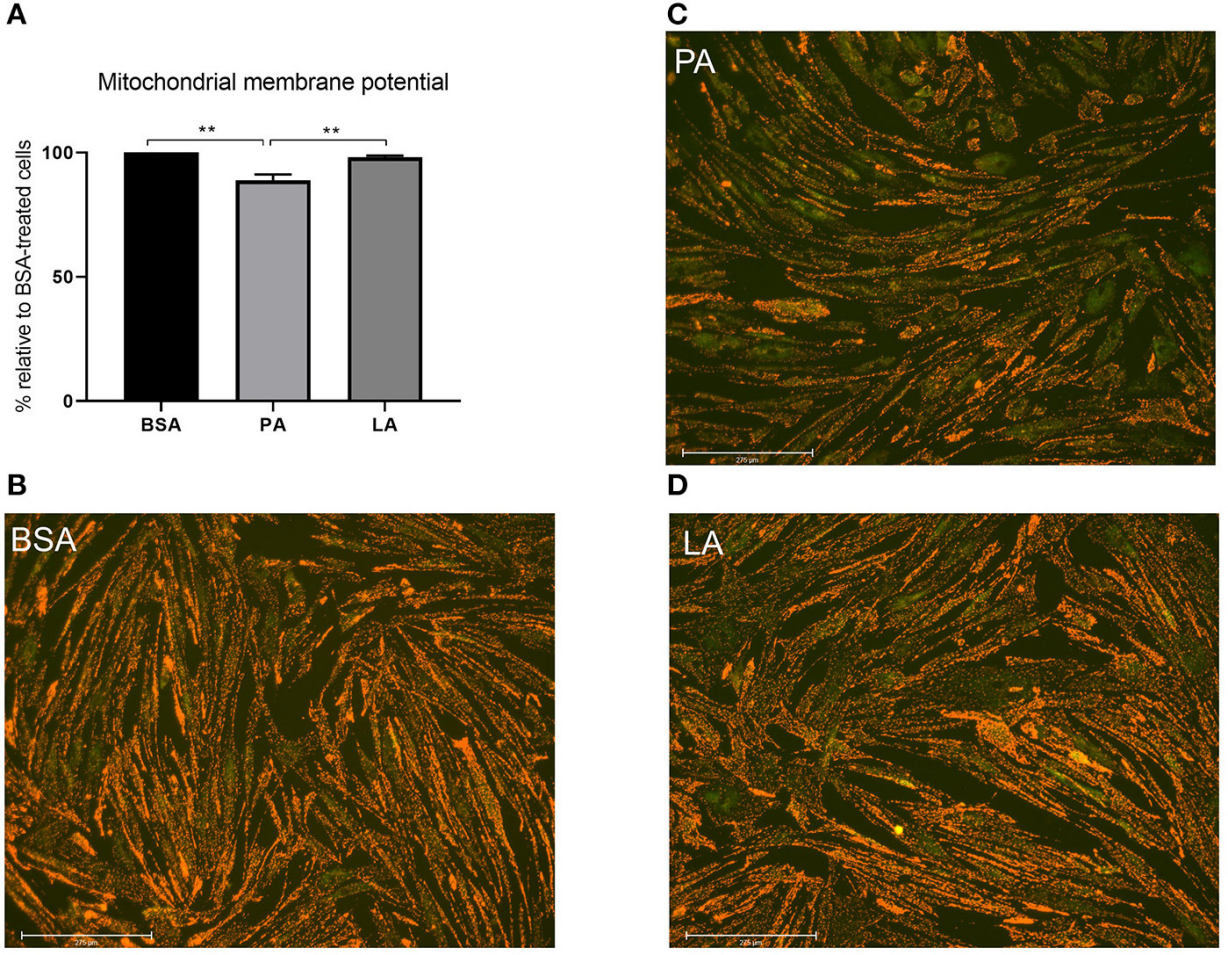
The impact of palmitic and lauric acid on mitochondrial membrane potential. **(A)** Mitochondrial membrane potential calculated as red to green JC-1 fluorescence ratio. Representative images of JC-1–derived red and green fluorescence of myotubes treated with BSA **(B)**, PA **(C)**, and LA **(D)**. Scale bar = 275 μM. Data are reported as means ± SEM, n = 4 cultures from independent donors (means of three replicates). **P < 0.01.

Considering mitochondrial dynamics being closely related to function (47), mitochondrial membrane potential was assessed to evaluate whether PA-induced mitochondrial fragmentation also resulted in a decrease in mitochondrial function. In agreement with its effect on mitochondrial fragmentation and downregulation of MFN-2, PA also caused a drop in mitochondrial membrane potential relative to myotubes treated with both BSA and LA (*p* < 0.01) (Figures 4A–D). Contrarily to PA, however, LA did not induce a drop in mitochondrial membrane potential relative to cells exposed to BSA alone (Figures 4A,B,D), indicating that the carbon-chain length, and not only the presence of double bonds, also plays an important role in dictating the impact of fatty acids on mitochondrial function.

## Discussion

The results of this study highlight the contrasting effects of PA and LA on metabolic inflammation, mitochondrial dynamics, and function in human primary myotubes, providing mechanistic insights on the effects of long- vs. medium-chain saturated fatty acids on metabolic health (31, 32) and particularly on insulin sensitivity (29, 50).

Metabolic inflammation, defined as a sterile inflammatory response (51) and typical of obesity and the metabolic syndrome (52), has been widely described for its ability to induce insulin resistance in metabolically active tissues (6, 51, 53, 54). Thus, the inability of LA to trigger an inflammatory response as opposed to PA may, at least in part, explain the different metabolic effects of medium- compared to long-chain saturated fatty acids (35, 55, 56). Particularly, the metabolic fate of PA and LA may be held responsible for their contrasting effects on inflammation. Indeed, LA compared to PA can directly enter the mitochondria for β-oxidation independently of carnitine palmitoyl transferase-dependent conjugation with carnitine (57), which therefore facilitate its catabolism. Increased fatty acid catabolism prevents the accumulation of lipotoxic lipid species, such as ceramides and diacylglycerols (32), which, in turn, has been shown to be, at least in part, responsible for long-chain saturated fatty acid-induced inflammation (14, 15). Furthermore, this confirms the inability of LA to trigger the activation of NFκB via the Toll-like receptor 4 (39).

Interestingly, PA and LA modulate the abundance of the mitochondrial electron transport chain complex proteins, suggesting that both fatty acids trigger adaptive responses aimed at increasing fatty acid catabolism. This notion, in particular, is supported by the induction of CIII-Core protein 2 (complex III). Indeed, there is a close physical and functional interaction between the mitochondrial electron transport chain supercomplexes and fatty acid oxidation enzymes (58, 59). Particularly, reducing equivalents resulting from the first reaction of the β-oxidation pathway in the mitochondrial matrix, catalysed by acyl-CoA dehydrogenases, are transferred to mitochondrial complex III, which directly interacts with electron flavoprotein dehydrogenase, the enzyme responsible for the oxidation of electron transfer flavoprotein (60). Thus, the induction of mitochondrial complex III in response to PA and LA challenge suggests myotubes mounting a response to cope with increased fatty acid availability. Nonetheless, despite promoting similar changes with regard to mitochondrial electron transport chain complex proteins, PA and LA differently impact upon mitochondrial dynamics with PA, contrarily to LA, downregulating MTF-2 and promoting mitochondria fragmentation. Importantly, a shift in mitochondrial dynamics towards fission is associated with a decrease in mitochondrial oxidative capacity (20) and therefore a compromised ability to effectively catabolise PA in excess. On the contrary, LA did not negatively impact mitochondrial dynamics maintaining a fused mitochondrial network, which, in turn, has been reported to promote fatty acid utilisation (21). This supports the notion that LA may be β-oxidised more effectively compared to PA (34), thereby preventing lipotoxicity and the downstream effects on skeletal muscle metabolic health. However, despite this being supported by previous evidence (34), it remains to be fully investigated by directly quantifying fatty acid β-oxidation (61) or tracking fatty acid metabolic fate by lipidomics (62). Furthermore, whereas LA did not impair mitochondrial membrane potential, exposure of myotubes to PA induced a drop in mitochondrial membrane potential, which further confirms that LA, compared to PA, preserved mitochondrial oxidative capacity as already reported in C2C12 myotubes (35). Nevertheless, despite assessing mitochondrial dynamics and mitochondrial membrane potential, which represent two key discriminants of mitochondrial function (20, 44, 47), a limitation of the present study is related to the fact that we did not directly assess mitochondrial oxidative capacity or oxygen consumption. Thus, the direct impact of PA and LA on mitochondrial oxidative capacity remains to be fully elucidated in human skeletal muscle. However, our data suggest that PA and LA differently regulate mitochondrial oxidative capacity, as demonstrated by mitochondrial fragmentation and the drop in mitochondrial membrane potential induced by PA.

The activation of the proinflammatory NFκB signalling in response to nutrient overload, including an excess of PA, has been recently reported to promote mitochondrial dysfunction, also underlaid by an increase in mitochondrial fission and a significant decrease in respiratory capacity (12). The same effect was reported herein, with PA inducing the activation of NFκB, which was paralleled by an increase in mitochondrial fragmentation and a drop in mitochondrial membrane potential, an effect completely absent in myotubes challenged with LA, albeit fatty acids being used at equimolar concentrations. Despite being tempting to blame metabolic inflammation for the effects of PA on mitochondria, the fact that metabolic inflammation may be a direct consequence of mitochondrial dysfunction rather than a cause must not be overlooked. Indeed, mitochondrial dysfunction in the face of increased PA availability favours the accumulation of ceramides, which, as already described, promote inflammation in metabolically active tissues (14). LA instead failed to trigger the activation of NFκB, which on the one hand may be responsible for the inability of this fatty acid to compromise mitochondrial health, but on the other may depend on LA failing to promote mitochondrial dysfunction and lipotoxicity (63, 64). Independently on whether mitochondrial fragmentation and the drop in mitochondrial membrane potential are a consequence or a cause of metabolic inflammation, these are both pathophysiological mechanisms associated with skeletal muscle insulin resistance, which, however, was not directly assessed in the experimental model used as part of this study. Therefore, the ability of PA to trigger these mechanisms, as opposed to LA, further supports the contrasting effects of these fatty acids on metabolic health, previously reported (29–33). Thus, despite PA and LA being both saturated fatty acids, their chain lengths, C12:0 vs. C16:0, respectively, appear to be an important discriminant in dictating the effects of these fatty acids on mitochondrial health as well as inflammation.

In conclusion, this study provides novel insights on the putative mechanisms responsible for the different metabolic outcomes of saturated fatty acids differing for their chain length (29, 30, 55, 65) in a physiologically relevant *in vitro* model closely mimicking human physiology: human primary myotubes. These findings support the possibility that the prevention of metabolic inflammation and mitochondrial dysfunction may be key mechanisms in preserving skeletal muscle insulin sensitivity in response to diets enriched in medium-chain triglycerides, as already demonstrated in rodents (50). Thus, LA, despite being a saturated fatty acid, did not induce the activation of pathophysiological mechanisms linked with insulin resistance, and therefore, eucaloric substitution of LA for PA may represent a valid strategy to preserve skeletal muscle metabolic health.

## Data Availability Statement

The raw data supporting the conclusions of this article will be made available by the authors upon reasonable request.

## Author Contributions

DS developed the hypothesis, performed the experiments, analysed results, and wrote the manuscript. DS, NO'C, and MA designed the experiments and coordinated the study. NL-M, NN, MA, and NO'C critically reviewed the manuscript and provided inputs on experimental procedures. All authors read and approved the final version of the manuscript.

## Conflict of Interest

The authors declare that the research was conducted in the absence of any commercial or financial relationships that could be construed as a potential conflict of interest.
